# Warranty for a better world? The politics of environmental knowledge in bioeconomic sustainability certificates

**DOI:** 10.1007/s13280-023-01836-1

**Published:** 2023-02-17

**Authors:** Håkon B. Stokland, Håkon Aspøy, Olve Krange, Ketil Skogen

**Affiliations:** 1 Norwegian Institute for Nature Research, Torgarden, Postbox 5685, 7485 Trondheim, Norway; 2 Norwegian Institute for Nature Research, Sognsveien 68, 0855 Oslo, Norway

**Keywords:** Bioeconomy, Certificates, Environmental knowledge, Politics of knowledge, Sustainability

## Abstract

Sustainability certificates are increasingly used as tools for shaping bioeconomic production processes and trade. However, their specific effects are subjected to debate. A multitude of certificate schemes and standards are currently in use, defining and measuring sustainability in the bioeconomy in highly varying ways. Different representations of environmental effects, resulting from the use of different standards or scientific methods in certification, can have very real implications for how, where and to which degree bioeconomic production can be conducted and the environment will be conserved. Further, the implications for bioeconomic production practices and management embedded in the environmental knowledge employed in bioeconomic sustainability certificates will produce different winners and losers, and privilege some societal or individual concerns at the expense of others. In this way, sustainability certificates share some characteristics with other standards and policy tools that embody political contingencies, but are presented and often understood as objective and neutral. The paper argues that the politics of environmental knowledge involved in these processes warrant more awareness, scrutiny and explicit consideration from decision makers, policy developers and researchers.

## Introduction

The utilization of renewable biological resources for products such as food, feed, timber and bioenergy is seen by many as an opportunity to supersede the era of fossil resources. This includes key political institutions such as the OECD and the EU (OECD 2009; EU 2018). In the more optimistic versions of this narrative, the bioeconomy promises to foster human health and nutrition and secure sustainable supplies of energy, water and raw materials, all while preserving soils, climate and the environment (Lindahl et al. 2017; Delbrück 2018; Asdal et al. 2021). However, this presupposes that bioeconomic production based on renewable resources is sustainable, in the sense that it can be conducted in a longer term without negative environmental or societal effects. Counter to some of the currently established narratives, sustainability is not an implicit result of the bioeconomy, and being based on renewable resources does not make the bioeconomy inherently sustainable (De Besi and McCormick 2015). Additionally, the expanding bioeconomy scenario implies a significant intensification in bioeconomic production, making the environmental impact equally more intensive (Sundnes et al. 2020; Rusch et al. 2022). Given that a significant part of ensuring sustainability in the bioeconomy is currently delegated to the use of certificate schemes and standards, rather than through legislation and government regulation (Angelstam et al. 2013; Löfmarck et al. 2017; Kleinschroth et al. 2019a, b), the role and effects of certificates and standards warrant scrutiny from decision makers and policy developers, as well as researchers.

In this paper, we first present the background of current bioeconomic sustainability certificates by shortly describing the history of certificate schemes in general, as well as the sustainability concept. Then, we draw on approaches that analyze the politics of environmental knowledge, and in particular the concept of ‘selective representations’ (Turnhout 2018), to discuss the emerging literature on bioeconomic sustainability certificates. Subsequently, we illustrate our argument with an example of a bioeconomic sustainability certificate scheme, namely the establishment of PEFC in Norway, before we conclude.

## Bioeconomic sustainability certificates

Certificates are often presented as powerful tools for shaping bioeconomic production processes and trade, but their specific effects are subjected to debate (Corsin et al 2007; Lytton 2014; Fouilleux & Loconto 2017; Loconto and Hatanaka 2018; Majer et al 2018). Being voluntary and often established by private organizations, they represent a form of regulation outside of government, or self-regulation. A multitude of certificates and related schemes are currently in use, aiming to improve the environmental sustainability of bioeconomic activity [e.g., USDA Organic, Carbon Footprint, The Program for the Endorsement of Forest Certification (PEFC), Forest Stewardship Council (FSC), American Tree Farm System (ATFS), The European Union Timber Regulation (EUTR), Sustainable Forestry Initiative (SFI), Aquaculture Stewardship Council (ACS), Global Aquaculture Alliance (GAA), Friends of the Sea (FoS), and Marine Stewardship Council (MSC)]. A number of such international certifications adapt their requirements to different national contexts, and, additionally, there exists a great number of national and local schemes. The complex certification landscape is continually changing with the development of new initiatives and schemes.

In a broader perspective, current certificates are a result of several developments in the twentieth century (Busch 2011). Most importantly, changes in commodity production, transportation and communication altered the relationship between producers, traders, and consumers. Food sold by the farmer at the market was replaced by food produced miles away, then transported, processed and packaged—involving a large number of people that the consumer never met. Trust rooted in stable and close relationships between seller and buyer on a local scale, could not be uphold in the modern, large-scale commodity chains (Zachmann and Østby 2011; Finstad 2013; Plasil et al. 2022; Stokland 2022). Further, governments have imposed extensive regulations on bio-production to safeguard public health and the environment, but the scope and effectivity of these regulations are frequently questioned. They do not guarantee public trust and will not necessarily help producers promote their commodities as particularly healthy or environmentally friendly.

Voluntary and privately managed certificates, labels and standards have become major tools employed to counteract the loss of trust in modern globalized commodity production, in recent decades also in relation to consumers’ trust in companies that claim to be sustainable and environmentally responsible (Lien and Nerlich 2004; Busch 2011; Zachmann 2011; Finstad et al. 2022). Such schemes are typically either stricter than government regulations, or they emphasize aspects of the products or the production process that regulations do not adequately cover. Interestingly, the relationship between voluntary certification schemes and government regulations can be quite complex or opaque. National legislation may even invoke private certification standards over which the state has no direct influence—as with the implementation of the PEFC standard for forestry in Norway,^1^ which we will discuss shortly.

Sustainability is a central concept for most bioeconomic certificate schemes that involve environmental aspects. This is not surprising, as it has become an omnipresent buzzword since the United Nations adopted the concept in the 1980s, and through UN initiatives such as the 1992 Rio Earth Summit, the 2005 Millennium Development Goals (MDGs), and the 2015 Sustainable Development Goals (SDGs) (Cardonna 2018). Sustainability is a standard feature of current public and political discourse, and almost any business of a certain size has pointed out which SDGs they are contributing to. Many different definitions have attempted to operationalize sustainability. The most prevalent definition still seems to be the one proposed by the Brundtland Commission: “development that meets the needs of the present without compromising the ability of future generations to meet their own needs” (World Commission on Environment and Development 1987). This definition is, however, so broad that it allows for countless interpretations when applied on a local and practical level (Dernbach 2003). Over time, sustainability has been conceptualized in multiple and shifting ways by different actors, and there has never been any real agreement on what constitutes sustainability (Robinson 2004; Cardonna 2014; Borowy 2018; Warde 2018; Mensah 2019; Purvis et al. 2019).

Parallel to the high-level international debates and the efforts of the UN system to achieve sustainability, a heterogeneous complex of sustainability standards and certificates has developed. In many ways, standards and certification schemes have become the leading governance mechanism for determining in practice what constitutes sustainability, and how to measure and assess it (Fransen 2015; Milder et al. 2015; Loconto and Hatanaka 2018). The prominent position granted to such instruments is related to a broader trend in natural resource governance towards voluntary agreements and self-regulation (Löfmarck et al. 2017). Certification schemes can be organized by independent institutions that develop standards for sustainability, and implementation is usually overseen by accredited third-party certifiers who audit compliance (Loconto and Busch 2010; Fouilleux and Loconto 2017; Loconto 2017). Schemes can, however, also be developed at the initiative of producers, for example through national or international trade organizations. Either way, sustainability certificate schemes attain much of their legitimacy from the use of standards and audits typically associated with apolitical, scientific, and objective methods (Porter 1995; Power 1997).

## The politics of environmental knowledge

Defining, measuring, and assessing sustainability in the bioeconomy is not a straight-forward matter, however. A substantial body of literature examines standards in their various forms (e.g., codes of conduct, guidelines, checklists, and regulations) and in numerous fields (e.g., electricity, food safety, healthcare, industry, information technology, and biodiversity) (Bowker and Star 1999; Miller and Rose 2008; Busch 2011; Stokland 2015, 2016). This literature has been expanded by an emerging research focus on sustainability standards and certificates. Some of these studies have attempted to improve current certificates, while others have been critical (Hatanaka 2010; Ransom et al. 2017; Loconto and Hatanaka 2018). In particular, the third-party model has been criticized for privileging quantitative and standardized knowledge over contextual and experience-based knowledge, thus also marginalizing ‘lay’ actors and producers of knowledge that do not employ strictly scientific-industrial production methods (Cheyns 2011; Ransom et al. 2017; Loconto & Hatanaka 2018).

Use of knowledge in sustainability certificates is, however, an issue that is not restricted to the privileging of scientific and expert knowledge over other forms of knowledge. The political implications embedded in the scientific knowledge used in certification schemes, and in particular in the choice of which scientific knowledge and methods to employ, warrant more scrutiny. It is a fundamental insight from social science that scientific and expert knowledge is neither objective nor neutral, opening the way for approaches such as ‘the social construction of scientific knowledge’ (Bloor 1976; Knorr-Cetina 1981), ‘post-normal science’ (Funtowicz and Ravetz 1993), ‘situated knowledges’ (Haraway 1988), and actor-network theory (Law and Hassard 1999; Latour 2005). There are many aspects and approaches in the wider social scientific literature on scientific knowledge that are potentially useful for studies of bioeconomic sustainability certificates. A thorough exploration of these is beyond the scope of this paper. Here, we focus on two aspects, namely what Esther Turnhout has termed ‘selective representations’ (2018), and the political implications embedded in them. This also means that we focus more on the politics of knowledge related to production and use of knowledge in certificates and standards, than on audits, verification and other control mechanisms that is usually built into them. The latter is an additional site for politics of knowledge, which has been more thoroughly examined elsewhere (Eden 2008; Konefal and Hatanaka 2011; Cook et al. 2016).

Scientific knowledge aims to represent reality by use of scientific methods, which among other things employs categories and classifications to order and make sense of the world (Latour 1987; Bowker and Star 1999). Although they often become naturalized and seemingly self-evident ways of understanding, such as the Red Lists representing threatened species (Jørstad & Skogen 2010; Stokland 2016), such categorizations and classifications are human made (Foucault 1970; Bowker and Star 1999). Further, scientific knowledge does not only represent reality, it is also constitutive of that very reality when its representations become naturalized or, in other words, what we take to be reality (Callon 2007; Law 2009). Due to the categorizations and classifications, which purpose is to simplify and make reality legible, scientific knowledge do not account for the full complexity of the matter in question (Scott 1998). Rather, some aspects of reality are foregrounded, while others are marginalized or ignored (Turnhout et al. 2007; Turnhout 2009). Therefore, the representation of reality resulting from specific scientific studies or expert assessments can best be described as partial, or ‘selective representations’, which focus on some aspects and leave others out (Turnhout 2018). Since different methods and indicators will produce selective representations that regularly differ, the choice of scientific method or approach will, to various degrees, be associated with different representations of reality. In the case of environmental knowledge employed in bioeconomic sustainability certificate schemes, the use of different standards, ways of measuring and auditing produce selective and different representations of the environmental effects of bioeconomic production.

The choice of categories, classifications and associated methods or indicators is, however, not merely an epistemological issue, in which the aim must be to arrive at the best possible representation of the environmental effects in question (Latour 2004; Turnhout 2018). The different, selective representations of the environmental effects of bioeconomic production embody different political implications. As we will illustrate, different representations of environmental effects can have very real and concrete implications for how, where and to which degree bioeconomic production can take place and at the same time protect the environment. Further, the implications for bioeconomic production practices and management embedded in the environmental knowledge employed in bioeconomic sustainability certificates will privilege some societal or individual concerns over others, and the end result is often closely tied to distribution of power. Thus, the politics of environmental knowledge refers to the political implications involved in the composition of scientific methods, definitions, categories, classifications and ways to measure and assess, in which some aspects of nature are foregrounded and others left out (Turnhout 2018).

## PEFC in Norway

In the case of establishing forest certification in Norway, the development of two different biodiversity mapping methodologies led to controversy over which one represented biodiversity most appropriately in relation to sustainable forestry. The prevalent certification scheme in Norwegian forestry is PEFC Norway, a branch of the international PEFC (Programme for the Endorsement of Forest Certification) organization. PEFC Norway was established in 1999, shortly after the initiation of the PEFC internationally. Until 2011 PEFC Norway was informed by the *Living Forest standard for sustainable forestry*. Resulting from a partnership begun in 1995 between parties representing environmental-, forestry-, and outdoor recreation interests, the Living Forest-standard was completed in 1998 (Levende skog, 1998). The original version stipulated 17 requirements for forestry to be certified as environmentally sustainable, whereas the revised version of 2006 contained 25 requirements (Levende skog 2006). However, in the 2010 revision the parties failed to reach an agreement on the use of potentially invasive alien tree species. Subsequently, negotiations broke down between environmental and outdoor recreation NGOs on the one hand, and forestry interests (predominantly forest owners’ organizations) on the other (Andresen 2013). The following year, in 2011, the Living forest-standard was abolished and succeeded by a PEFC Norway standard after ratification from PEFC (PEFC Norway 2011). Since then, forest certification in Norway has been undertaken by the forestry sector itself, formally overseen by private certifiers which are appointed by forest owners’ organizations.^2^

Although the sustainable forest certification scheme evoked controversy from an early stage, the criticism escalated with the resignation of the environmental interest groups from the scheme. In the following, we focus on one of the requirements of the standards that has been subjected to considerable debate, namely that of efforts to maintain overall biodiversity through conserving woodland key habitats.

In 1992, preceding the Living forest collaboration by three years, a group of conservation biologists introduced a methodology for biodiversity mapping in Norway’s boreal forests. The group, called *Last Chance* (‘Siste sjanse’), was part of Friends of the Earth Norway’s local branch in Oslo and Akershus. Their methodology was heavily inspired by the activities of the Swedish group *A step ahead* (‘Steget före’) whose core belief was that biodiversity in productive forests could be more or less maintained by refraining from logging certain biodiverse-rich areas. The task, then, was to locate such areas. Last Chance issued several handbooks and reports, instructing mappers on how to perform such mapping (Siste sjanse 1993; Haugset et al. 1996; Løvdal et al. 2002). In sum, these constituted a distinctive methodology that came to be known as the Siste sjanse-method (SiS). The publications of Last Chance largely focused on explaining how areas of particular importance for biodiversity could be located. Referred to as *woodland key habitats*, these were to be identified according to various criteria, such as red-listed species and species indicative of old-growth forests.

SiS required mappers to be educated in conservation biology and thus competent to make assessments in the field. In fact, the methodology relied on them to perform a practice termed biological discretion. Last Chance claimed that while classification could be useful, nature in many cases was too complex for standardized methodologies to sufficiently locate biologically rich areas. Therefore, a specific task in the procedure to identify woodland key habitats was dedicated to the mappers’ own evaluation and judgement. After identification, the habitats would be plotted into maps to help forest owners and others in forestry management to avoid future loss of biodiversity.

With the introduction of the Living forest-standard in 1998 came a requirement devoted specifically to the preservation of woodland key habitats. The standard stated that mapping had to be carried out and that the environmental values of the identified woodland key habitats had to be documented and maintained. With regards to size, the standard stipulated that forest properties below 50 hectares had to attain a minimum of 0.5 hectares, and that for larger forest properties 1% of the surface had to be maintained. However, the standard also noted that for the moment, mapping should be based on current methodology, until the results from initiated research projects were ready (Levende skog 1998).

During the same year, the Ministry of Agriculture allocated funds directly to the Norwegian Institute for Forest Research (NISK), earmarked for the research project Environmental Inventories in Forests (EiF). According to the Ministry, a more scientifically sound methodology was needed for biodiversity mapping in forests (The Ministry of Agriculture 1998; Kløvstad 2015). In many respects, the resulting EiF-methodology resembled that of SiS. Both aimed to locate biodiverse areas through fieldwork and to protect these when conducting forestry. Both also made use of the concept of woodland key habitats. In fact, the two would often be lumped together under the common designation of woodland key habitat-registration. However, there were important differences in how the two methodologies conceptualized and identified such habitats, or, in other words, in how they enacted ‘selective representations’ of the forests in question.

The question of descriptive versus normative tasks, and thus distinguishing mapping from management, was central to the differences between SiS and EiF. With the launch of EiF, the leader of the research project stated that previous registration practices had failed to distinguish between them. Alluding to SiS, he argued that this mapping methodology had wrongfully assumed that woodland key habitats could simply be found. This was not the case, he claimed. Such habitats were rather to be considered as measures to prevent biodiversity loss, a view that was also expressed in the Living forest-standard (Levende skog 1998). Woodland key habitats had little to do with registering biodiversity and more to do with decision making, in this view (Gjerde 2000). Although SiS was not mentioned explicitly in this case, the Ministry’s Forestry director later launched a similar criticism directly against SiS in an interview with a forestry journal (Kløvstad 2015). The leader of the EiF-project also asserted that red-listed species and other biodiversity occurrences had proven to be less concentrated than what had previously been expected, claims that later would be utilized by forestry companies to defend EiF against objections from Last Chance and environmental NGO’s (Gjerde 2000; Bøhn 2007). In line with the project’s observations, the EiF handbook expressed only a moderate belief in the effectiveness of preserving woodland key habitats to prevent biodiversity loss. Rather, it suggested that other measures, such as preserving single occurrences of biodiversity, often small habitats such as dead trees, could be more appropriate (Baumann and Gjerde 2002). EiF also embodied a different logic for determining if and how occurrences of biodiversity constituted areas that were worthy candidates for woodland key habitat status. In contrast to the use of discretion in SiS, occurrences were counted in EiF and standardized limits for each geographical region were set to determine satisfactory levels of biodiversity concentrations. Hence, EiF and SiS would often yield divergent representations of woodland key habitats in particular areas, according to the ‘selections’ exercised in the composition of their methodologies.

The Ministry’s Forestry director, in the EiF handbook’s foreword, emphasized that decisions were up to the forest owners themselves and that mapping would not compromise their alternatives, but rather the opposite. He further noted that the EiF-process presumed that forest economy would be taken into account, too, when assessing which areas should be logged or preserved (Ekanger 2002).

Although the Ministry had no formal roles in the Living forest-standard and PEFC, their involvement in EiF was decisive. Another crucial incident happened in 2004, when the Ministry revised the regulations for subsidies to environmental measures under the Forestry Act. Whereas the regulations of 2001 also accepted “other similar scientifically documented methods”, those of 2004 accepted only EiF (The Norwegian Government 2001, 2004). The changes delegitimized SiS and more or less put an end to its use in a forestry context. Several environmental organizations objected to the process with little success (Norges Naturvernforbund 2003; SABIMA 2003). The controversy reached a climax when the National Committee for Research Ethics in Science and Technology (NENT) launched an investigation of the Ministry after allegations that it had violated the ethical standards for research funding. Eventually, the investigation ruled in favor of the Ministry (NENT 2004; Gulbrandsen 2008).

A further twist in this case is the power granted to the PEFC standard in Norwegian legislation. The Forestry Act, which is under the domain of the Ministry for Agriculture and not the Ministry for Environment, includes regulations setting out rudimentary environmental requirements for logging and other forestry activities. Interestingly, the regulations refer explicitly to the requirements of the PEFC standard, albeit in a general and opaque way (§4 and 5, FOR-2006-06-07-593 2006). However, Norwegian authorities have no role in the development of the PEFC standard, and no jurisdiction over certified parties’ compliance with its requirements. In fact, the scope of the legal connection between the act and the PEFC standard has never been settled and remains a legal “grey zone”. But the fact remains that the state opts to enforce environmental protection requirements in forestry through a private certification scheme—a scheme over which the state has no direct influence.

Thus, the SiS and EiF methodologies for identifying woodland key habitats, although similar in many respects, embodied different political implications in terms of what would be required of forestry practices under PEFC. In their critique of SiS, proponents of EiF insinuated that SiS mappers exerted too much influence through their use of non-standardized and qualitative assessments of areas worthy of being designated woodland key habitats, and thus preservation. It was also required in SiS that mappers should be educated in conservation biology, so this is indeed a question of which actors are granted an opportunity to influence the size and location of areas that should be preserved under PEFC. In EiF, on the other hand, the standardized mapping practices left mappers with little flexibility and little opportunity to exercise conservation biology competence. However, insisting that woodland key habitats were not simply existing in the forests, but rather measures to be taken, EiF granted forest owners with more flexibility in making decisions about the size and location of areas to be preserved under PEFC than SiS (Aspøy and Stokland 2022). The political implications of the environmental knowledge in this case, therefore, was mainly related to how much flexibility forest owners were granted in deciding the size and location of areas to be preserved under PEFC, and to which degree conservation biologists could influence decisions. The political struggles related to the application of the two methodologies under PEFC, and the political force exerted by the Ministry of Agriculture in ensuring that EiF prevailed over SiS, is indicative of the importance of these political implications. Currently, Norwegian regulations of sustainable forestry refers to the PEFC standard rather than making its own requirements, and this standard only accepts EiF as a mapping methodology for identifying areas that should be preserved when logging. Therefore, we can conclude that the politics of environmental knowledge in this case ended up favoring forestry concerns over environmental ones.

## Conclusion

Because of the real-world effects on production processes, humans and the environment, the construction of environmental sustainability certificates in the bioeconomy should be considered an activity with political aspects. Since the environmental knowledge employed by the certification schemes constitute selective representations, in which different definitions and ways to measure and assess sustainability implicate different winners and losers, the production of environmental knowledge for bioeconomic standards and certificates should also be considered an activity with political aspects. In this way, sustainability certificates and the associated production of environmental knowledge share some characteristics with other standards and policy tools that embody political contingencies, but are presented and often understood as objective and neutral.

We have shown an example of this in the case of the sustainable forest certification scheme PEFC in Norway. Two competing methodologies for mapping biodiversity-rich areas, which were to be preserved during logging, was developed in relation to the initiation of this standard. The two methodologies had different political implications, and, in the end, it was specified in the PEFC standard that the one favoring forestry concerns over environmental ones, which was also exclusively supported by the Ministry of Agriculture, should be employed exclusively in the identification of areas to be preserved.

Based on this, we argue that scrutinizing the politics of environmental knowledge needs to go beyond critical investigations of how quantitative and standardized knowledge is often privileged over contextual and experience-based knowledge. This is not to say that the marginalization of ‘lay’ actors and producers that do not employ strictly scientific-industrial production methods has become less important in the development and practices of bioeconomic sustainability certificate schemes. It means, however, that the politics involved in scientific knowledge and standards, both regarding the composition of specific methods and approaches, and the prioritization and marginalization of different schemes, additionally warrants more scrutiny.

When making decisions and developing policies, governments and other decision makers should be more aware of and explicitly consider the political implications of certificate schemes and the environmental knowledge used in them. These implications will often favor some actors and societal concerns over others, and hence impact the effects and potential achievement of the goals of decisions and policies. There is also reason to discourage governments and decision makers from assuming that sustainability certificate schemes and standards are objective instruments, which perform regulation on bioeconomic production outside of public government institutions in a politically neutral way.

## Data Availability

Not applicable.

## Footnotes

1 Another example include a regulation in Gabon which made FSC certification compulsory for forest concessions by 2022 (Karsenty 2019).

2 FSC (Forest Stewardship Council) certification has had limited use in Norway, but is now expanding, and a Norwegian version has recently been accepted by FSC. Internationally, the FSC certification scheme has been a site for methodological disagreements paralleled by those we describe here for PEFC in Norway, in particular in relation to the efforts of FSC to conserve intact forest landscapes on global scale (Kleinschroth et al. 2019a, b).
